# Effectiveness and Safety of Mechanical Debridement for Treating Experimental Peri-Implantitis in Elderly Rats Receiving Oncological Dosages of Zoledronate

**DOI:** 10.3390/ijms27031355

**Published:** 2026-01-29

**Authors:** Luan Felipe Toro, Eduardo Quintão Manhanini Souza, Vinícius Franzão Ganzaroli, Jéssica de Oliveira Alvarenga Freire, Leandro Lemes da Costa, Estevão Lopes Pereira, Beatriz Alexandrelli Machado, João Martins de Mello-Neto, Mariza Akemi Matsumoto, Cláudio Aparecido Casatti, Luciano Tavares Ângelo Cintra, Letícia Helena Theodoro, Valdir Gouveia Garcia, Edilson Ervolino

**Affiliations:** 1 Department of Basic Sciences, School of Dentistry, São Paulo State University (UNESP), Araçatuba 16015-050, SP, Brazil; luan.toro@unesp.br (L.F.T.); eduardoquintao@hotmail.com (E.Q.M.S.); vinicius.ganzaroli@unesp.br (V.F.G.); jessica.freire@unesp.br (J.d.O.A.F.); leandro.lemes@unesp.br (L.L.d.C.); estevao.pereira@unesp.br (E.L.P.); beatriz.alexandrelli@unesp.br (B.A.M.); mariza.matsumoto@unesp.br (M.A.M.); claudio.casatti@unesp.br (C.A.C.); 2 Institute of Biosciences, São Paulo State University (UNESP), Botucatu 18618-000, SP, Brazil; 3 Department of Basic Subjects, Marília Medical School (FAMEMA), Marília 17519-030, SP, Brazil; 4 College of Medicine and Dentistry, James Cook University, Cairns, QLD 4870, Australia; joao.martinsdemelloneto@jcu.edu.au; 5 Department of Restorative Dentistry, School of Dentistry, São Paulo State University (UNESP), Araçatuba 16015-050, SP, Brazil; luciano.cintra@unesp.br; 6 Department of Diagnostic and Surgery, School of Dentistry, São Paulo State University (UNESP), Araçatuba 16015-050, SP, Brazil; leticia.theodoro@unesp.br; 7 Latin American Institute of Dental Research and Education (ILAPEO), Curitiba 80810-030, PR, Brazil; valdir.garcia@unesp.br

**Keywords:** debridement, dental implant, osteonecrosis, peri-implantitis, zoledronate

## Abstract

This study evaluated the effectiveness and safety of mechanical debridement (MD) in treating experimental peri-implantitis (EPI) in rats with osseointegrated implants, specifically those treated with high-dose zoledronate. Senescent Wistar rats underwent the extraction of their upper incisor, followed by immediate implant placement. After 8 weeks, the implants were exposed, and a transmucosal component was placed. The animals were divided into four groups: Control (C), ZOL, ZOL-EPI, and ZOL-EPI-MD. In the 9th week, drug treatment commenced, consisting of the administration of 0.45 mL of a vehicle (for group C) or zoledronate (for groups ZOL, ZOL-EPI, and ZOL-EPI-MD) every 4 days over 10 weeks. After 5 weeks of drug treatment, a cotton bandage was placed around the implants to induce EPI in the ZOL-EPI and ZOL-EPI-MD groups. In the ZOL-EPI-MD group, the ligature was removed at week 16, and local treatment was performed using MD. Euthanasia was conducted at week 19. Histological sections were obtained and stained with hematoxylin–eosin for histopathological and histometric analyses, such as the percentage of total bone tissue (B.Ar/T.Ar) and the percentage of non-vital bone tissue (NVB.Ar/B.Ar). Immunohistochemical reactions were performed to detect TNFα, IL-1β, VEGF, OCN, and TRAP. In the peri-implant connective tissue, mild, intense, and moderate inflammatory infiltrates were observed in the ZOL, ZOL-EPI, and ZOL-EPI-MD groups, respectively. Immunolabeling for TNFα and IL-1β correlated with these histopathological findings. The ZOL and ZOL-EPI-MD groups showed lower immunolabeling for VEGF compared to the control group. There was a reduction in TRAP-positive cells and lower immunolabeling for OCN in the groups treated with zoledronate, with the ZOL-EPI-MD group displaying even lower levels of OCN compared to the ZOL group. While there was no significant difference in B.Ar/T.Ar across the groups, both the ZOL, ZOL-EPI, and ZOL-EPI-MD groups exhibited higher levels of NVB.Ar/B.Ar, with the ZOL-EPI-MD group showing the highest NVB.Ar/B.Ar compared to ZOL and the other groups. In conclusion, MD, as a standalone treatment, showed neither effectiveness nor safety in the management of EPI in rats that received high doses of zoledronate.

## 1. Introduction

Medication-related osteonecrosis of the jaw (MRONJ) is a pathological condition characterized by the presence of exposed bone in the maxillofacial region, or bone that can be probed through an intraoral or extraoral fistula, for a period greater than eight weeks, in patients undergoing previous or current treatment with antiresorptive and/or antiangiogenic drugs, and who have no history of radiotherapy or metastatic disease in this region [1].

A recently identified type of MRONJ associated with dental implants (DIs) is known as MRONJ-DI. Clinical studies indicate that MRONJ-DI can occur early in about one-third of patients, while the remaining two-thirds may experience it as a late complication. When MRONJ-DI develops early, it is typically associated with the surgical procedures involved in placing DIs [2,3]. When it appears later, its onset is related to the presence of an already osseointegrated implant or the conditions associated with it [2,4,5,6,7,8,9,10]. Systematic reviews and clinical studies indicate that MRONJ-DI occurs more frequently in patients receiving oncological dosages of antiresorptive drugs, especially when this treatment begins after the DI has already osseointegrated and is functioning properly in the oral cavity [11,12].

It has been recommended that DIs be contraindicated for patients undergoing treatment with these oncological doses of antiresorptive drugs [13] to help prevent the development of MRONJ-DI. However, patients with osseointegrated implants who later require treatment with antiresorptive drugs raise significant concerns, as they make up the majority of cases of MRONJ-DI [14]. One of the concerning clinical consequences of MRONJ-DI is the involvement of large areas in the maxilla or mandible, often leading to pathological fractures in these bones. While Otto et al. (2011) [15] reported pathological fractures in about 3% of patients with post-extraction MRONJ, Escobedo et al. (2020) [5] found that this condition affects around 60% of individuals with MRONJ-DI. This significant discrepancy underscores the severe impact of this condition on patients’ quality of life. It can hinder or even render rehabilitation impossible, often resulting in long-term complications that affect the entire stomatognathic system.

In addition to the risks associated with drug treatment and the presence of a DI, local risk factors such as chronic inflammatory-infectious processes have been identified as direct contributors to the development of MRONJ-DI processes [1]. In this context, several studies have indicated a strong correlation between peri-implantitis (PI) and the occurrence of MRONJ-DI in patients who have used or are currently using antiresorptive drugs [3,11,16,17,18]. Peri-implantitis is a pathological condition primarily initiated by bacteria but is significantly influenced by local and systemic factors. It affects the tissues surrounding the DI and is characterized by inflammation and progressive loss of supporting bone tissue, which can ultimately lead to the loss of the DI [19].

Epidemiological data suggest that 1–47% (mean of 22%) of patients with a DI experience PI, which may initially be presented as mild or even asymptomatic. Furthermore, smokers or patients with a prior history of periodontitis have a higher incidence of PI [19]. As the condition advances, deeper probing depths, the formation of peri-implant pockets, bone loss, pus drainage, pain, swelling, and recession of the peri-implant mucosa are observed [19,20,21,22]. In addition to potentially leading to the loss of DIs in patients undergoing treatment with antiresorptive drugs, PI may also represent a significant local risk factor for the development of MRONJ-DI, resulting in more severe consequences. Therefore, early diagnosis and treatment are essential for the success of DI rehabilitation therapy and for preventing the onset of MRONJ-DI [11,17,18].

Due to the complex nature of its pathogenesis and the multiple factors that can contribute to the progression of this condition, various clinical protocols have been developed and adopted as therapeutic options for PI. These protocols encompass non-surgical approaches, conservative treatments, regenerative techniques, resective surgeries, and combinations of various methods [23,24]. However, there are currently no universally accepted guidelines within the scientific community. As a result, the clinical experience of healthcare professionals plays a crucial role in determining the appropriate course of action [23,24,25].

The choice of treatment for PI generally depends on the severity and extent of the disease. Mechanical debridement (MD) is considered the standard and first-line treatment for the majority of PI cases [3,26]. MD, performed with manual dental instruments, has several advantages over other treatment methods. These advantages include greater technical simplicity, lower costs, shorter treatment times, and reduced morbidity [27]. Additionally, studies have shown that MD as monotherapy can effectively manage PI, particularly when employed during the early phases [27,28]. It is worth noting that during therapy with antiresorptive drugs, more invasive procedures, such as surgical treatment, should be avoided [1,13]. In this situation, treating PI is effective as it prevents disease progression and can maintain the osseointegrated DI’s function. Furthermore, the treatment is safe as it does not increase the risk of MRONJ or its serious complications/consequences. However, the limited number of studies examining the efficacy and safety of MD for PI during antiresorptive therapy makes it challenging to establish an evidence-based approach, making its evaluation in standardized experimental models necessary.

This study hypothesizes that MD, being a minimally invasive therapy, may prove to be both effective and safe in managing PI and reducing the risk of MRONJ-DI. Therefore, the aim of this study was to assess the effectiveness and safety of MD in treating experimental peri-implantitis induced by ligature in osseointegrated implants within the maxilla of ageing rats, which were subsequently given oncological doses of zoledronate.

## 2. Results

### 2.1. Assessment of the Animals’ Overall Health and Clinical Examination of Their Oral Condition

The animals exhibited good overall health throughout the experiment and tolerated the medication protocol, as well as the surgical, clinical, and anesthesia procedures. In an individual analysis, all animals showed a higher body weight at the beginning of the experiment (prior to the extraction and installation of the implant platform) compared to the day of reopening and exposing the implant platform. Despite this, good adaptation to the surgical procedures and the diet of crushed feed was observed, leading to subsequent weight gain. Both intragroup and intergroup analyses indicated no significant differences in the average body weight of the animals during the three measurement points.

During the preoperative intraoral clinical examination, no changes were noted in the teeth or oral cavity of the animals. At the time of reopening and exposure of the implant platform, the implants were found to be osseointegrated, stable, and resistant to both digital pressure and the installation of transmucosal components. The peri-implant soft tissues appeared to be entirely healed and normal. In the intraoral clinical examination conducted just prior to euthanasia, all implants remained clinically stable. However, the animals in the groups treated with zoledronate (ZOL, ZOL-EPI, and ZOL-EPI-MD) exhibited marked redness in the peri-implant soft tissues when compared to the control group (group C). The ZOL-EPI group showed a significant accumulation of biofilm associated with the cotton ligature, and more severe oedema and erythema were observed around the implants. The animals in the ZOL-EPI-MD group presented a clinical picture similar to that of the ZOL group, but without the presence of biofilm or the significant oedema seen in the ZOL-EPI group. Clinically, no lesions resembling established MRONJ were observed in any of the animals.

### 2.2. Histopathological Characteristics of Peri-Implant Tissues

**Figure 1:**
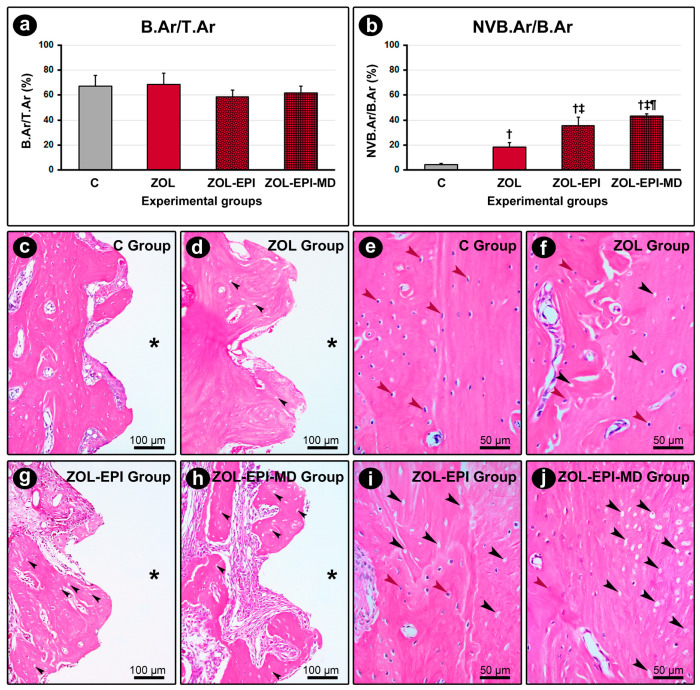
Percentage of total bone tissue (B.Ar/T.Ar), percentage of non-vital bone tissue (NVB.Ar/B.Ar) and histopathological appearance of peri-implant bone tissue: (**a**) graph showing the B.Ar/T.Ar for groups C, ZOL, ZOL-EPI, and ZOL-EPI-MD; (**b**) graph showing the NVB.Ar/B.Ar for groups C, ZOL, ZOL-EPI, and ZOL-EPI-DM; (**c**–**j**) photomicrographs showing the quantity and pattern of cellularity and structuring of the bone tissue located between the implant coils in groups C (**c**,**e**), ZOL (**d**,**f**), ZOL-EPI (**g**,**i**), and ZOL-EPI-DM (**h**,**j**). Statistical test: Shapiro–Wilk test and variance analysis (ANOVA) followed by the Tukey post-test. Abbreviations and symbols: †, statistically significant difference in relation to C; ‡, statistically significant difference compared to ZOL; ¶, statistically significant difference compared to ZOL-EPI; *, space previously occupied by the implant coils; black arrows, gaps devoid of osteocytes; red arrows, osteocytes. Staining: HE. Original magnification: 200× (**c**,**d**,**g**,**h**) and 400× (**e**,**f**,**i**,**j**). Scale bars: 100 μm (**c**,**d**,**g**,**h**) and 50 μm (**e**,**f**,**i**,**j**).

In group C, a dense, non-modeled connective tissue was observed around the DI. This tissue contained a large number of fibroblasts, with few inflammatory cells and high levels of vascularization. The surrounding bone tissue exhibited a cellular pattern and structure that appeared normal. There was no evidence of peri-implant bone loss or active bone resorption near the alveolar bone crest. The osteoclasts present displayed typical morphological characteristics and were distributed diffusely throughout the area (Figure 1c,e).

The ZOL, ZOL-EPI, and ZOL-EPI-MD groups all exhibited connective tissue surrounding the DI with varying levels of inflammatory infiltrate: mild in the ZOL group, moderate in the ZOL-EPI group, and intense in the ZOL-EPI-MD group. The inflammatory infiltrate primarily consisted of mononuclear cells. Notably, in the ZOL-EPI group, there was an extracellular matrix characterized by significant fibrous involvement, a limited number of blood vessels, and an inflammatory infiltrate that extended into the surrounding bone tissue.

In the ZOL group, the bone tissue surrounding the implants was comparable to that of group C; however, the amount of non-vital bone tissue was significantly higher. Additionally, no peri-implant bone loss was observed in this group (Figure 1d,f). In the ZOL-EPI and ZOL-EPI-MD groups, bone loss around the implants was observed, specifically in the cervical third. These groups also exhibited a higher amount of non-vital bone tissue. Notably, in the ZOL-EPI-MD group, the areas of non-vital bone were not only more numerous but also much more extensive compared to the other groups (Figure 1g–j). In the ZOL, ZOL-EPI, and ZOL-EPI-MD groups, there was a significantly lower number of osteoclasts compared to group C. The few osteoclasts present in these groups were rounded, hypernucleated, larger, and distanced from the bone matrix—characteristics that indicate cellular inactivity.

### 2.3. Percentage of Total Bone Tissue (B.Ar/T.Ar) and Percentage of Non-Vital Bone Tissue (NVB.Ar/B.Ar) in the Peri-Implant Region

There was no difference in B.Ar/T.Ar between the C, ZOL, ZOL-EPI, and ZOL-EPI-MD groups (Figure 1a). However, the ZOL, ZOL-EPI, and ZOL-EPI-MD groups showed higher NVB.Ar/B.Ar compared to the C group. Additionally, the ZOL-EPI group had higher NVB.Ar/B.Ar than the ZOL group, and the ZOL-EPI-MD group exhibited even higher NVB.Ar/B.Ar compared to both the ZOL and ZOL-EPI groups (Figure 1b).

### 2.4. Immunolabeling for TNFα, IL-1β, VEGF, and OCN in Peri-Implant Tissues

**Figure 2:**
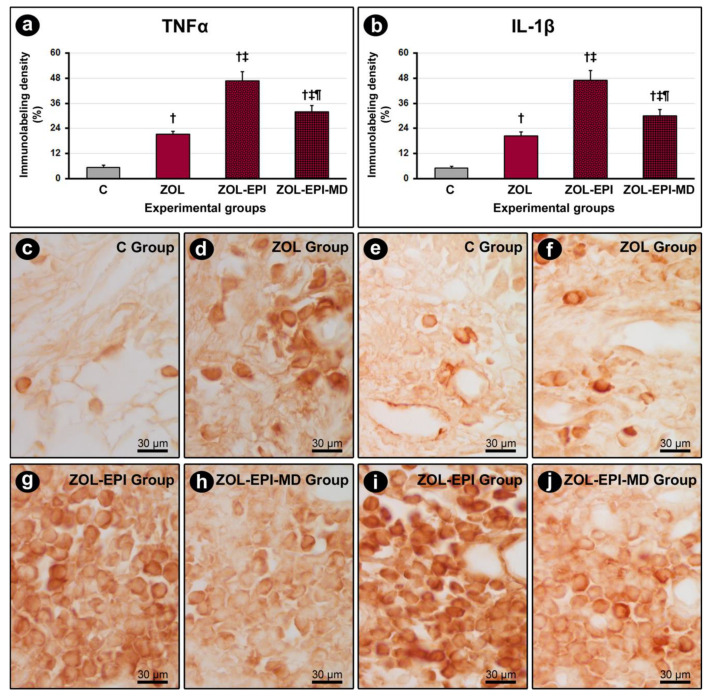
Immunolabeling for TNFα and IL-1β in peri-implant connective tissue: (**a**) graph showing the immunolabeling density for TNFα in the peri-implant connective tissue of groups C, ZOL, ZOL-EPI, and ZOL-EPI-MD; (**b**) graph showing the immunolabeling density for IL-1β in the peri-implant connective tissue of groups C, ZOL, ZOL-EPI, and ZOL-EPI-MD; (**c**–**j**) phototomicrographs showing the immunolabeling pattern for TNFα in the peri-implant connective tissue of groups C (**c**), ZOL (**d**), ZOL-EPI (**g**), and ZOL-EPI-MD (**h**), and the immunolabeling pattern for IL-1β in the peri-implant connective tissue of groups C (**e**), ZOL (**f**), ZOL-EPI (**i**), and ZOL-EPI-MD (**j**). Statistical test: Shapiro–Wilk test and variance analysis (ANOVA) followed by the Tukey post-test. Abbreviations and symbols: †, statistically significant difference in relation to C; ‡, statistically significant difference compared to ZOL; ¶, statistically significant difference compared to ZOL-EPI. Original magnification: 100×. Scale bar: 30 μm.

Immunolabeling for TNFα and IL-1β was predominantly observed in inflammatory cells and, to a lesser extent, in the extracellular matrix of the peri-implant connective tissue (Figure 2c–j). The groups treated with ZOL, ZOL-EPI, and ZOL-EPI-MD exhibited a higher density of immunolabeling for TNFα and IL-1β compared to the control group (group C). Furthermore, the ZOL-EPI and ZOL-EPI-MD groups showed a higher density of immunolabeling for these cytokines compared to the ZOL group. However, the ZOL-EPI-MD group had a lower density of immunolabeling for TNFα and IL-1β compared to the ZOL-EPI group (Figure 2a,b).

**Figure 3:**
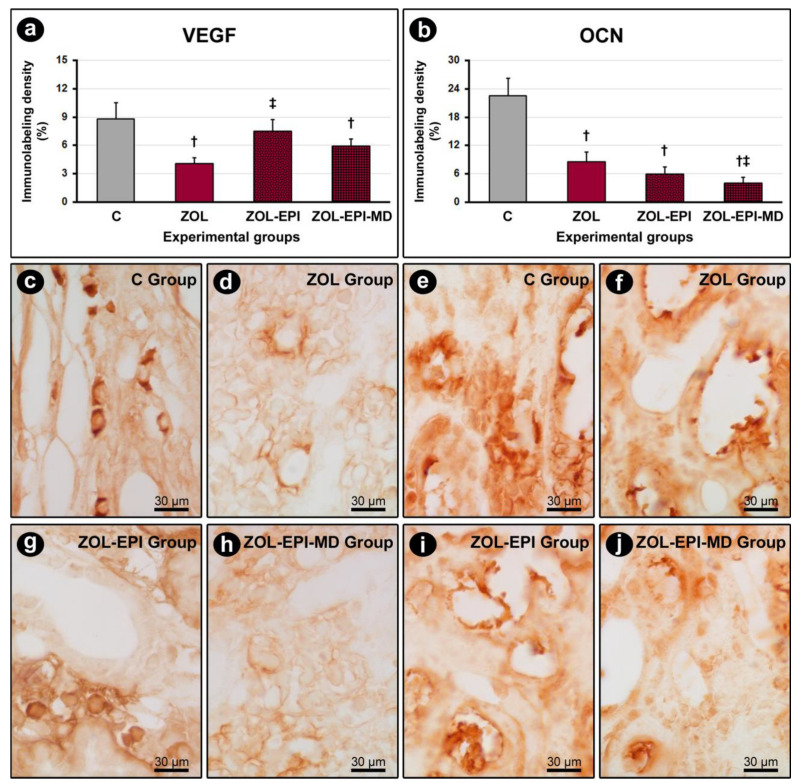
Immunolabeling for VEGF in peri-implant connective tissue and immunolabeling for OCN in peri-implant bone tissue: (**a**) graph showing the immunolabeling density for VEGF in the peri-implant connective tissue of groups C, ZOL, ZOL-EPI, and ZOL-EPI-MD; (**b**) graph showing the immunolabeling density for OCN in the peri-implant bone tissue of groups C, ZOL, ZOL-EPI, and ZOL-EPI-MD; (**c**–**j**) phototomicrographs showing the immunolabeling pattern for VEGF in the peri-implant connective tissue of groups C (**c**), ZOL (**d**), ZOL-EPI (**g**), and ZOL-EPI-MD (**h**), and the immunolabeling pattern for OCN in the peri-implant bone tissue of groups C (**e**), ZOL (**f**), ZOL-EPI (**i**), and ZOL-EPI-MD (**j**). Statistical test: Shapiro–Wilk test and variance analysis (ANOVA) followed by the Tukey post-test. Abbreviations and symbols: †, statistically significant difference in relation to C; ‡, statistically significant difference compared to ZOL. Original magnification: 100×. Scale bar: 30 μm.

For VEGF, immunolabeling was primarily present in fibroblasts and, to a lesser degree, in the extracellular matrix of the peri-implant connective tissue (Figure 3c,d,g,h). The ZOL and ZOL-EPI-MD groups demonstrated a lower density of immunolabeling for VEGF compared to the control group (group C). In contrast, the ZOL-EPI group displayed a higher density of immunolabeling for VEGF compared to the ZOL group (Figure 3a).

In the peri-implant bone tissue, immunolabeling for OCN was mainly found in osteoblasts and, to a lesser extent, in the bone matrix (Figure 3e,f,i,j). The ZOL, ZOL-EPI, and ZOL-EPI-MD groups exhibited a lower density of immunolabeling for OCN compared to the control group (group C). Additionally, the ZOL-EPI-MD group showed a lower density of immunolabeling for OCN compared to the ZOL group (Figure 3b).

### 2.5. Immunolabeling for TRAP in Peri-Implant Bone Tissue

**Figure 4:**
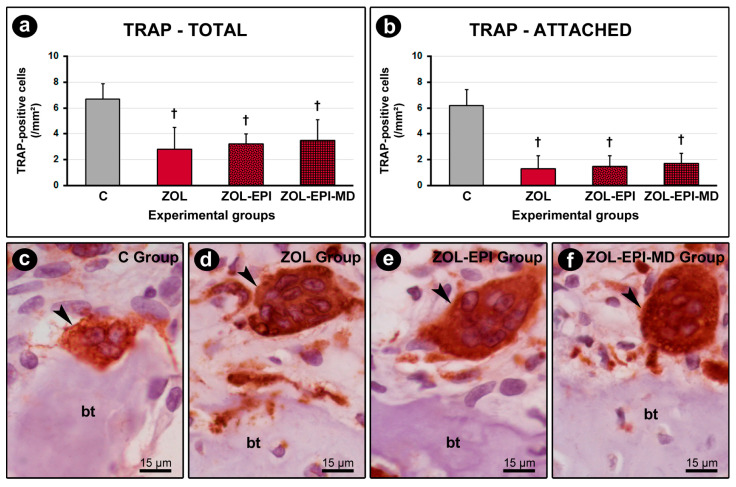
Immunolabeling for TRAP in peri-implant bone tissue: (**a**,**b**) graphs showing the total number of TRAP-positive cells (**a**) and the number of TRAP-positive cells attached to the bone matrix (**b**) per mm^2^ in the peri-implant bone tissue of groups C, ZOL, ZOL-EPI, and ZOL-EPI-MD; (**c**–**f**) photomicrographs showing the immunolabeling pattern for TRAP in the peri-implant bone tissue of groups C (**c**), ZOL (**d**), ZOL-EPI (**e**), and ZOL-EPI-MD (**f**). Statistical test: Shapiro–Wilk test and variance analysis (ANOVA) followed by the Tukey post-test. Abbreviations and symbols: †, statistically significant difference in relation to C; bt, bone tissue; black arrows, immunolabeling TRAP-positive multinucleated cells. Counterstain: Harris hematoxylin. Original magnification: 1000×. Scale bar: 15 μm.

Immunolabeling for TRAP was detected in osteoclasts (Figure 4c–f). The ZOL, ZOL-EPI, and ZOL-EPI-MD groups exhibited a lower number of TRAP-positive cells, both total and those attached to the bone matrix, compared to group C (Figure 4a,b).

## 3. Discussion

In recent years, a specific type of MRONJ associated with DIs has been identified, referred to as MRONJ-DI. Most cases of MRONJ-DI occur as a late complication related to DIs. This condition is concerning, particularly because many patients who have undergone rehabilitation with DIs in recent decades are ageing and may eventually experience skeletal-related events that require treatment with antiresorptive drugs. Several studies have reported a strong correlation between the presence of PI and the occurrence of MRONJ-DI in patients who have used or are currently using antiresorptive medications [3,11,16,17,18]. Our research group has developed an experimental model in rats where DIs are placed at the extraction site of the upper incisor. After achieving osseointegration and modelling of peri-implant bone, we initiated treatment with zoledronate. During this treatment, we induced experimental peri-implantitis (EPI) by placing a cotton ligature around the implant neck. Our findings indicate that EPI triggers significant peri-implant inflammation, but minimal peri-implant bone loss; however, a notable increase in the amount of non-vital bone tissue surrounding the DI is noted, which predisposes this area to MRONJ-DI.

Experimental animal models can assist in elucidating the etiopathogenesis and in proposing or evaluating treatments for MRONJ-DI. This study employed an experimental model that integrated the primary clinical conditions associated with both the patient and the disease. Senescent rats were used to reflect well-established epidemiological risk factors, such as female sex and advanced age, and were treated with zoledronate, the most potent nitrogen-containing bisphosphonate [29]. The dose and treatment regimen were adapted from protocols used in oncological therapy in humans, as documented in a previous study, given the higher incidence of MRONJ [29,30]. We posit that models based on epidemiological characteristics enhance both the reliability of the data and its translational relevance. In this study, we aimed to evaluate the effectiveness and safety of non-surgical treatment of EPI. Our results indicated that MD, when used as a monotherapy, was neither an effective nor a safe treatment for EPI in ageing rats that were administered an oncological dose of zoledronate. Inflammation, especially when it is exacerbated or prolonged, is often associated with the occurrence of MRONJ [31,32,33]. Histopathological analysis revealed the presence of inflammatory infiltrates in the tissues surrounding the titanium implants in all groups treated with zoledronate (ZOL, ZOL-EPI, and ZOL-EPI-MD). In contrast, the control group (C), which also had osseointegrated implants but did not receive systemic treatment with zoledronate, showed no such inflammation. Furthermore, we observed a higher density of immunolabeling for TNFα and IL-1β—two important cytokines known for their pro-inflammatory activity—in the groups treated with zoledronate, compared to the control group.

Bisphosphonates (BPs) are primarily known for targeting osteoclasts, but their effects extend beyond these cells. BPs also affect various cell types, including immune cells, leading to disruptions in both innate and acquired immune responses. This, in turn, creates an imbalance in the expression of cytokines with pro-inflammatory and anti-inflammatory activities [34]. In particular, BPs directly affect the polarization and activation of macrophages, altering the balance between M1 and M2 macrophages [35,36,37]. M1 macrophages are mainly pro-inflammatory, capable of phagocytosing and digesting various pathogens, as well as serving as antigen-presenting cells [36]. Conversely, M2 macrophages are considered anti-inflammatory and promote the resolution of inflammation, removal of apoptotic cells, and facilitate tissue repair and remodeling [36]. Studies indicate that BPs encourage the polarization of M1 macrophages while inhibiting M2 polarization. This imbalance leads to an increase in pro-inflammatory mediators, which in turn attract and activate more M1 macrophages and other immune cells [34,35,38]. Consequently, the actions of BPs on these immune cells lead to an exacerbation and prolongation of the inflammatory response, which may result in tissue damage and hinder the transition to the resolution phase.

The increase in the magnitude of the inflammatory response and elevated levels of pro-inflammatory cytokines are histopathological and molecular parameters that strongly correlate with the occurrence of MRONJ-like lesions in various experimental models [39,40,41,42]. Research indicates that knockout mice lacking the cytokines TNFα, IL-1α/β, and IL-6 demonstrated resistance to the development of osteonecrotic lesions, even when treated with high doses of bisphosphonates (BPs) [10,41]. This finding further underscores the significant role of BP-induced inflammation in the pathogenesis of this condition.

In this study, the inflammatory condition was significantly worsened in the ZOL-EPI and ZOL-EPI-MD groups, likely due to the introduction of additional local risk factors in these animals. Among all groups, ZOL-EPI exhibited the highest intensity and extent of the local inflammatory response, characterized by elevated levels of TNFα and IL-1β. These are typical indicators consistent with the progression of the induced inflammatory-infectious condition. The significant accumulation of microbial biofilm around the cotton ligature during disease progression, along with the resulting dysbiosis of the resident microbiota, allowed pathogenic microorganisms to proliferate rapidly and invade deeper tissues [43]. The presence of bacterial toxins, particularly endotoxin LPS from Gram-negative bacteria, triggers self-amplifying responses by stimulating the activation of M1 monocytes and macrophages, leading to the production of more inflammatory mediators [44,45]. Moreover, LPS and other pore-forming exotoxins can activate inflammasomes, which are cytosolic multiprotein complexes found in cells of the granulocytic lineage. This activation results in the release of various cytokines. Other exotoxins may function as superantigens, prompting T lymphocytes to release additional cytokines [45].

While a definitive consensus has not yet been reached regarding whether MRONJ is triggered or sustained by an infectious process, it is established that infection occurs in areas of necrosis. Bacteria are commonly found in MRONJ samples [46]. The presence of infection contributes to the ongoing inflammatory condition and delays the healing process, effectively restarting and perpetuating the cycle. Therefore, the link between chronic inflammation, impaired tissue repair, and increased susceptibility to infection in tissues previously weakened by BPs may position PI as a contributing factor to the development of MRONJ.

Following the administration of MD and the subsequent removal of bacterial deposits and the primary plaque retention factor (the cotton ligature), the ZOL-EPI-MD group exhibited a relative reduction in the intensity of the inflammatory infiltrate observed in the peri-implant connective tissue at 21 days post-treatment. This was accompanied by lower levels of TNFα and IL-1β compared to the group that did not receive local mechanical therapy. However, the characteristics of tissue inflammation were still more pronounced in the ZOL group and especially in group C. Despite MD being performed manually using curettes and instruments that may inadvertently traumatize tissues around the implant—potentially leading to an initial increase in the inflammatory response and raising the risk of MRONJ—it is reasonable to conclude that after effective debridement of the peri-implant area, which includes the removal of a significant amount of bacterial contamination, toxins, tissue remnants, and immune cells, there would be a noticeable reduction in both the intensity and extent of the inflammatory process.

Zoledronate functions by targeting the mevalonate pathway. It inactivates the enzyme farnesyl-PP synthase, which prevents the production of crucial molecules such as farnesyl-PP and geranylgeranyl-PP. These molecules are essential for the prenylation of small GTPases, which play a crucial role in various cellular processes in osteoclasts, including membrane morphological changes and cytoskeletal rearrangements necessary for bone resorption. Additionally, these processes are involved in the intracellular trafficking of vesicles and the prevention of premature apoptosis in osteoclasts [34,47,48]. In this study, even though the analysis area was relatively small compared to the entire organism, we observed that treatment with zoledronate produced the expected pharmacological effects on osteoclasts. This was evident from the reduced number of TRAP-positive cells, their distinctive morphological features, and the tissue characteristics noted in specimens from the treated groups. In the ZOL, ZOL-EPI, and ZOL-EPI-MD groups, the existing osteoclasts were observed as large, rounded, hypernucleated cells, often located away from the bone matrix, indicating typical signs of inactivity [49]. Moreover, reflecting the inactivation of these cells, the histometric analysis revealed no significant bone loss around the implants in groups where EPI was induced. This finding is significant as it relates to the disease’s progression, which likely would have resulted in bone loss had antiresorptive drug treatment not been administered.

Ruggiero et al. (2022) [1] clinically defined MRONJ as the presence of exposed bone or bone that can be probed through an intraoral or extraoral fistula in the maxillofacial region. They also proposed various stages of disease progression. Specifically, stage 0 occurs before the clinical manifestation of MRONJ, where radiographic and/or histopathological signs of bone necrosis can be detected, even in asymptomatic patients. Although the present study employed an experimental model, we can still relate this information to our findings. The histopathological evaluation, further supported by histometric analysis data, revealed numerous areas of non-vital bone tissue in the peri-implant region. This was especially evident in the groups treated with zoledronate, with the most pronounced effects observed in the ZOL-EPI group, and even more so in the ZOL-EPI-MD group.

The osteonecrotic lesions observed in this study primarily fall into the “frozen type” (type I), as classified by Kwon et al. (2014) [3]. This type is characterized by the presence of bone necrosis surrounding the implant and in the adjacent alveolar bone. Additionally, there is inflammation in the peri-implant soft tissues, variable degrees of inflammatory infiltrate in the medullary spaces, and extensive areas of Haversian bone tissue lacking cellular components. It is important to note that this classification was based solely on the histopathological aspects of the peri-implant tissues, as there were no clinical manifestations resembling MRONJ-DI in this experimental rat model across the different groups.

The inflammation induced by BPs around osseointegrated implants, as previously described, seems to be directly related to the increase in areas of non-vital bone tissue in the peri-implant region. Preclinical and clinical studies suggest that the onset of MRONJ is influenced by a combination of systemic risk factors, such as the use of potent antiresorptive and/or antiangiogenic drugs, and local risk factors, including tooth extractions and/or infectious/inflammatory conditions, such as periodontitis and osteonecrosis. Local risk factors associated with inflammation and/or infection lead to the production of pro-inflammatory cytokines, which exacerbate the inflammatory process and activate signaling pathways that result in the programmed cell death of osteocytes [50].

Under normal circumstances, after bone resorption in the area, apoptotic bodies of osteocytes are swiftly removed by adjacent phagocytes. However, with BP therapy, the resorptive activity of osteoclasts is reduced, leading to a significantly slower or even inhibited removal of non-vital bone tissue. Consequently, osteocytes that are undergoing apoptosis or are in an advanced stage of autophagy may remain within the lacunocanalicular network of the bone matrix for extended periods. This increases the likelihood of secondary necrosis occurring in that region [50].

In addition to osteocyte apoptosis induced by inflammation, another distinct form of programmed cell death called necroptosis has been identified. Unlike apoptosis, necroptosis involves the activation of specific intracellular pathways, resulting in cell death that displays characteristics of accidental damage. This type of cell death is marked by the rupture of the plasma membrane, leading to the leakage of cytoplasmic contents and damage-associated molecular patterns (DAMPs) into the surrounding extracellular matrix [50,51,52,53]. Various stimuli and chemical agents can trigger necroptosis in osteocytes, including inflammatory cytokines, damaged DNA, lipopolysaccharides (LPS), and pathogen-associated molecular patterns (PAMPs) [52,54]. DAMPs released during necroptosis or from secondary necrosis in apoptotic cells contribute to amplifying the inflammatory response, which raises the levels of pro-inflammatory cytokines. These cytokines can then activate intracellular signaling pathways that lead to the apoptosis or necroptosis of additional osteocytes, perpetuating this cycle. Therefore, it is reasonable to propose that the severity of the local inflammatory process is directly proportional to the extent of non-vital bone tissue. This aligns with the findings of the current study, which explains the increased amount of non-vital bone tissue observed in the ZOL-EPI and ZOL-EPI-MD groups.

In the present study, ZOL-EPI-MD exhibited the highest amount of non-vital bone tissue in the peri-implant region compared to the other experimental groups. Although this finding does not have a clear connection to the observed decrease in inflammatory parameters within this group, it can be explained by the mechanical trauma inherent in the procedure and the different phases of tissue repair: inflammatory, proliferative, and maturation [55]. MD causes trauma to the tissues where it is applied. Additionally, by removing significant bacterial deposits and plaque retention factors that contribute to disease progression, it promotes the repair of affected tissues. This combination of mechanical disruption and tissue repair triggers an acute inflammatory response immediately after the procedure. This intense initial inflammatory reaction may lead to the death of osteocytes, resulting in bone necrosis. The reabsorption of non-vital bone tissue does not occur due to the suppression of osteoclastic activity caused by BPs. As the tissue repair process progresses, the severe inflammation initially observed in the soft tissues tends to diminish and transition into the maturation phase [55]. However, the consequences at the bone level persist and may even worsen, suggesting that conventional MD monotherapy can be considered a risk factor for MRONJ-DI.

In vitro studies [56,57,58] have shown that zoledronate, at high concentrations, exerts potent cytotoxic effects on pre-osteoblasts and osteoblasts. Huang et al. (2016) [56] demonstrated that zoledronate has dose-dependent inhibitory effects on the expression of type I collagen, alkaline phosphatase, and osteocalcin (OCN) in osteoblasts. Additionally, it reduces the differentiation of precursor cells by decreasing the levels of bone morphogenetic protein 2. Furthermore, zoledronate has been shown to significantly hinder the differentiation of adipose tissue-derived stem cells into osteoblasts [59]. Consistent with the in vitro findings, the immunohistochemical analysis conducted in this study revealed lower immunolabeling for OCN in groups treated with zoledronate compared to the control group (group C). This potential cytotoxic effect of high doses of BPs on osteoblastic lineage cells could extend to osteocytes, which are interconnected with osteoblasts through cytoplasmic processes. In addition to possible direct cytotoxicity, significant alterations in cellular dynamics may lead to severe atrophy of osteocytes and even their death [50]. This culminates in the accumulation of empty lacunae that are not effectively reabsorbed due to the pharmacological effects of BPs. In this context, the ZOL-EPI-MD group, which showed the highest amount of non-vital bone tissue in the peri-implant region, also exhibited a lower density of immunolabeling for OCN compared to the ZOL group. This observation underscores the intrinsic correlation between the two characteristics analyzed.

Another local factor that can harm peri-implant tissues is the antiangiogenic effect of BPs. This was demonstrated in our study, which showed lower levels of VEGF immunolabeling in animals treated with zoledronate (ZOL) and the ZOL-EPI-MD group. Supporting these results, animals that underwent tooth extraction while receiving an oncological dose of zoledronate also exhibited reduced VEGF labelling during the alveolar bone repair process [60]. Wehrhan et al. (2011) [61] observed a decrease in angiogenesis and the formation of new blood vessels in the mucoperiosteal connective tissue of patients treated with zoledronate or pamidronate. Additionally, Wang et al. (2022) [62] reported a reduction in vascular density, as well as in the average perimeter and diameter of blood vessels in soft tissues adjacent to areas of bone necrosis in patients diagnosed with medication-related osteonecrosis of the jaw (MRONJ). It is important to note that the higher density of VEGF immunolabeling in the ZOL-EPI group compared to the ZOL group may be attributed to the severe inflammatory response in this group, which significantly stimulates angiogenesis and increases local vascularization. Thus, it is increasingly clear that osteonecrosis lesions and the underlying pathophysiology of MRONJ result from a complex interplay of various cellular and tissue factors, rather than from a single cause or event.

PI is a challenging pathological condition that often leads to unfavorable outcomes. The current study revealed that during treatment with zoledronic acid, the progression of PI—without any local intervention—can lead to significant inflammation of the peri-implant tissues. This inflammation is associated with the development of numerous areas of non-vital bone tissue in the surrounding region, along with various cellular-level alterations. These findings suggest that advancing PI can result in MRONJ-DI, potentially leading to the loss of osseointegrated implants and more serious complications. Conversely, when PI is treated locally through manual methods, as traditionally recommended, it can exacerbate the accumulation of non-vital bone tissue around the implants. This indicates that such local therapies may not be effective or safe in these specific local and systemic conditions. Therefore, dentists face a critical challenge in managing PI in patients undergoing treatment with antiresorptive drugs since the progression of the disease can lead to MRONJ-DI, and the most frequently employed local treatments may also contribute to this risk.

Hence, to prevent MRONJ and the loss of DIs in patients undergoing treatment with BPs, primary prevention of PI is crucial. Patients should be thoroughly informed about the potential risks associated with their condition and encouraged to schedule regular dental appointments, ideally before starting their medication protocol. Dental professional should determine this frequency. At the first sign of inflammation around the implant sites, the dentist should utilize minimally invasive treatment methods to manage the condition effectively and ideally promote regression, preventing further progression to the deeper surrounding tissues. If PI is already present, prompt and adequate treatment remains essential. Given the unsatisfactory results observed in this study with the use of monotherapy, it is important to explore and evaluate alternative peri-implant therapeutic strategies.

## 4. Materials and Methods

### 4.1. Animals Used and Ethical Considerations

This study utilized 28 female Wistar rats (*Rattus norvegicus*), aged 20 months and weighing an average of 400 g. The animals were sourced from the Central Animal Facilities of São Paulo State University, School of Dentistry (FOA-UNESP). They were housed in plastic cages within the experimental vivarium, with unrestricted access to water and food. The environment maintained a 12-h light/dark cycle, a room temperature of 22 ± 2 °C, a ventilation system providing 20 air changes per hour, and a relative humidity of 55 ± 5%. All animal handling procedures adhered to the standards set by the National Council for Animal Control and Experimentation (CONCEA) and complied with the ARRIVE guidelines (https://arriveguidelines.org). This experimental protocol received approval from the Ethics Committee on Animal Use (CEUA) at FOA-UNESP (REF: FOA 00330–2019).

### 4.2. General Anesthesia

For surgical procedures and other interventions that may cause pain and discomfort and require immobilization for an extended period and euthanasia, the animals were anesthetized using an intramuscular (IM) injection of ketamine hydrochloride at a dosage of 80 mg/kg (Syntec do Brasil Ltd.a., São Paulo, Brazil) in combination with xylazine hydrochloride at a dosage of 10 mg/kg (Syntec do Brasil Ltd.a.).

### 4.3. DI and Transmucosal Component Design

**Figure 5:**
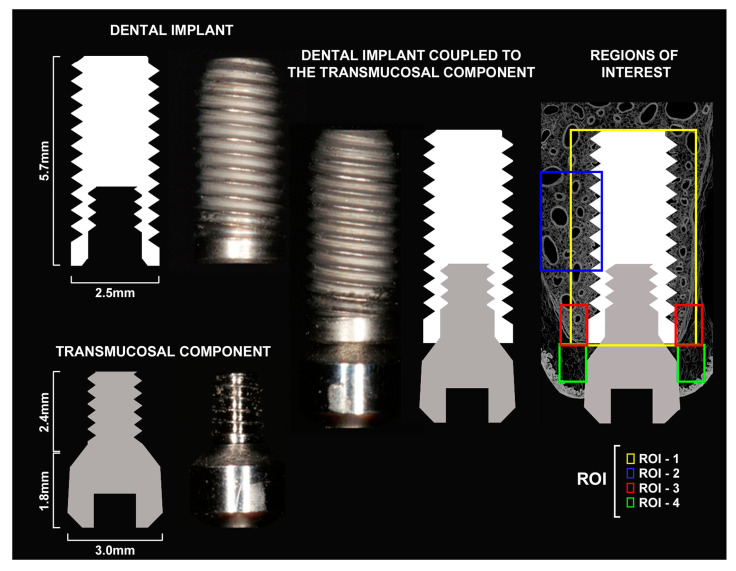
Schematic drawing and image of the titanium dental implant structure and the transmucosal component, individualized with their respective dimensions, and coupled together. Regions of interest (ROI) for histopathological, histometric, and immunohistochemical analyses in the different peri-implant tissues.

The titanium dental implant used was cylindrical, measuring 2.5 mm in diameter and 5.7 mm in length, with an internal hexagon connection. Its surface had been treated through sandblasting and acid etching (Dentfix^®^, Allied Titanium Eireli, São Paulo, Brazil). The transmucosal component measured 1.8 mm in length and 3.0 mm in diameter, containing a 2.8 mm portion with spirals that allow it to be attached to the implant platform (Dentfix^®^, Allied Titanium Eireli) (Figure 5).

### 4.4. Extraction of the Upper Right Incisor

**Figure 6:**
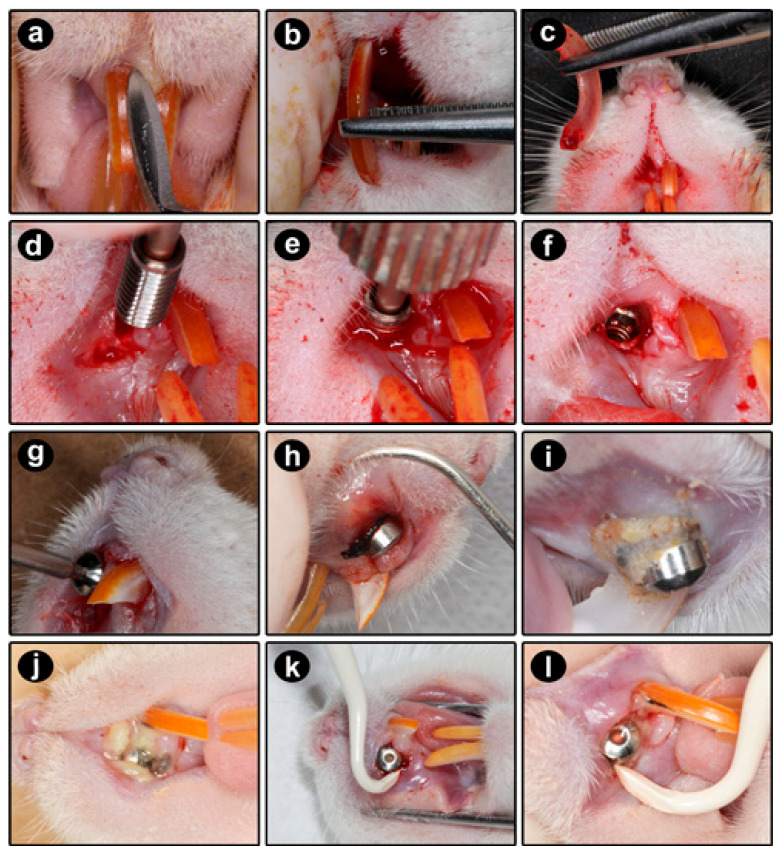
Sequence of surgical and clinical procedures performed throughout the experimental period: (**a**–**c**) syndesmotomy and luxation, apprehension, and extraction of the right upper incisor; (**d**–**f**) installation of the dental implant in the alveolus previously occupied by the right upper incisor, through threading movements with a digital driver attached to the internal connection of the implant, followed by suturing of the peri-implant soft tissues; (**g**,**h**) installation and positioning of the cotton ligature around the cervical portion of the transmucosal component attached to the implant to induce experimental peri-implantitis; (**i**–**l**) accumulation of biofilm around the cotton ligature two weeks after its installation and use of plastic manual curettes to perform mechanical debridement in the animals in the ZOL-EPI-MD group.

On the first day of the experimental period, the upper right incisor was extracted. The animals were anesthetized, and both extraoral and intraoral antisepsis were performed using topical solutions of 10% polyvinylpyrrolidone iodine (PVPI) and 0.12% chlorhexidine digluconate, respectively. Specialized dental instruments were used to carefully and minimally traumatize the periodontal tissues during the extraction process, which included syndesmotomy and luxation of the upper right incisor (Figure 6a). Blunt-tipped clinical forceps were used to grasp the tooth in the anatomical neck region, followed by rotational traction [63] to complete the extraction (Figure 6b,c).

### 4.5. Installation of the DI

Immediately after the extraction of the upper right incisor, a DI was placed in the alveolus. The insertion of the implant was carried out slowly and gradually using a digital driver. Threading movements were employed until adequate primary stability was achieved in a subgingival position (Figure 6d–f). After this, the soft tissues were repositioned and sutured using 4.0 silk thread. To prevent infections, the animals were administered 0.01 mL/100 g of a polyvalent anti-infective combination (Zoetis Indústria de Produtos Veterinários Ltd.a., São Paulo, Brazil) via intramuscular injection. To minimize biomechanical stress in the area and facilitate feeding, the animals were provided with crushed feed throughout the experiment. Additionally, the lower incisors were filed weekly to prevent injuries at the site of the DI.

### 4.6. Reopening, Exposure of the DI Platform, and Installation of the Transmucosal Component

In the 8th week, after anesthetizing the animals and ensuring antisepsis, we performed a subperiosteal infiltration using 2% lidocaine with 1:100,000 adrenaline (DFL Indústria e Comércio S.A., Rio de Janeiro, Brazil) to facilitate local hemostasis. We then made a linear incision along the alveolar ridge above the implant region and folded back the flap to expose the DI connection platform. At this stage, we clinically assessed the secondary stability of the implant and installed a transmucosal component onto the platform, using a digital screwdriver for the threading movements (Figure 6g). Finally, we sutured the tissues around the exposed transmucosal component with a simple stitch using 4.0 silk thread to reapproximate and stabilize the area in the oral cavity [63].

### 4.7. Experimental Groups and Drug Protocol

**Figure 7:**
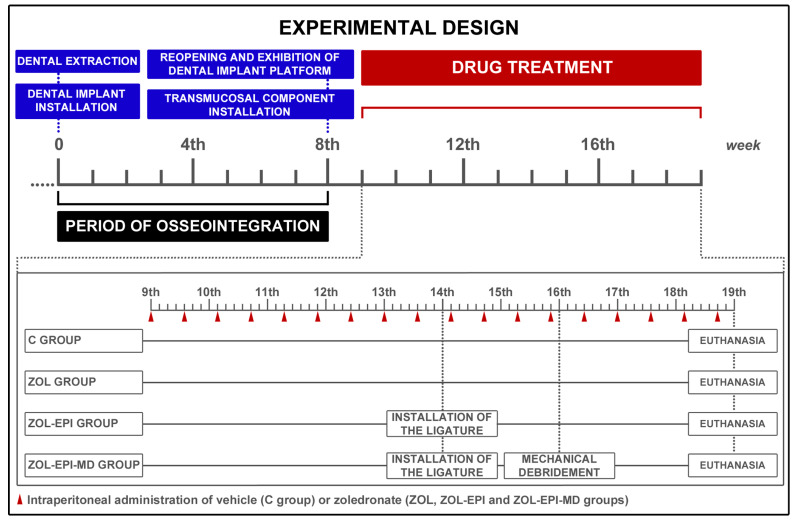
Diagram illustrating the main procedures performed throughout the experimental period, highlighting the events included during the drug treatment plan and the different experimental groups (C, ZOL, ZOL-EPI, and ZOL-EPI-DM).

In the 9th week of the experimental period, each animal was assigned a sequential number from 1 to 28. Using Minitab^®^ 17 software (Minitab Inc., State College, PA, USA), the animals were randomly distributed into four groups: C (Control) (*n* = 7), ZOL (*n* = 7), ZOL-EPI (*n* = 7), and ZOL-EPI-MD (*n* = 7) at the start of the drug protocol. The animals in the control group (C) received 0.45 mL of physiological saline solution (NaCl 0.9%) via intraperitoneal (IP) injection every 4 days for a total of 10 weeks, continuing until the 19th week. The other groups also received 0.45 mL of the physiological saline solution, but it was supplemented with 100 μg/kg of zoledronate (Sigma-Aldrich Co., St. Louis, MO, USA) via IP injection every 4 days for the same duration (Figure 7). The dosage and treatment plan were based on previous studies and are in accordance with the protocol currently used to complement cancer therapy in humans, adapted for use in rats [30,42,60,63,64].

### 4.8. Ligature-Induced Experimental Peri-Implantitis (EPI)

At week 14, after the animals were anesthetized and antiseptic procedures were followed, a cotton ligature (Coats Corrente Ltd., São Paulo, Brazil) was placed around the cervical portion of the transmucosal component in the peri-implant sulcus region for the ZOL-EPI and ZOL-EPI-MD groups (Figure 6h). The purpose of this procedure was to facilitate the accumulation of bacterial biofilm [63] and trigger an epithelial inflammatory response (EPI) (Figure 6i,j). In the ZOL-EPI group, the ligature remained in place for the duration of the experiment. In contrast, the ZOL-EPI-MD group had the ligature in place for only 2 weeks, after which it was removed, and local treatment was administered.

### 4.9. Local Treatment Through Mechanical Debridement

In the 16th week, after the animals had been anesthetized and antisepsis had been performed, the cotton ligature in the ZOL-EPI-MD group was removed. A single MD session was conducted using plastic manual curettes (Supremo^®^ Instrumentos Cirúrgicos, São Paulo, Brazil). This was characterized by mesiodistal and vestibulopalatal traction movements applied to the exposed DI coils, with a focus on the spaces between them and the area where they are connected to the transmucosal component (Figure 6k,l).

### 4.10. Euthanasia, Sample Collection, and Processing

At week 19, the animals were anesthetized and euthanized through transcardiac perfusion with 100 mL of physiological saline solution containing 0.1% heparin. This was followed by fixation using an 800 mL solution of 4% formaldehyde (Sigma-Aldrich) in phosphate-buffered saline (PBS) at a concentration of 0.1 M and a pH of 7.4. The maxillae containing the implants were dissected and underwent post-fixation for 48 h in the same fixative solution. Subsequently, they were subjected to demineralization in a solution of 10% ethylenediaminetetraacetic acid (EDTA) (Sigma-Aldrich) in PBS for 75 days.

### 4.11. Histological Processing

After complete demineralization, DIs were carefully unscrewed and removed using a digital wrench attached to the internal connection of the transmucosal components installed in the implants. The samples were then dehydrated in ethanol, cleared in xylene, and impregnated with histological paraffin for embedding. Histological sections, each with a thickness of 4 μm, were obtained from the medial to the distal end, following the long axis of the space previously occupied by the implant. The sections corresponding to the central portion of the DI were collected on silanized slides (Platinum Line^®^ StarFrost, Mercedes Medical LLC, Lakewood Ranch, FL, USA). One part of the sections was set aside for staining with hematoxylin and eosin (HE) for histopathological and histometric analyses, while the other part was designated for immunohistochemical reactions.

### 4.12. Immunohistochemical Reaction

Immunohistochemical reactions were conducted using the indirect immunoperoxidase technique, following the protocols established by Souza et al. (2024) [63] and Ervolino et al. (2019) [64]. Histological slides were divided into five batches, each incubated for 24 h with one of the following primary antibodies at a concentration of 1:100: rabbit anti-tumor necrosis factor (TNFα) antibody (orb11495; Biorbyt, Cambridge, UK), rabbit anti-interleukin 1-beta (IL1-β) antibody (orb382131; Biorbyt), rabbit anti-vascular endothelial growth factor (VEGF) antibody (orb191500; Biorbyt), mouse anti-osteocalcin (OCN) antibody (sc365797; Santa Cruz Biotechnology, Dallas, TX, USA), and mouse anti-tartrate resistant acid phosphatase (TRAP) antibody (sc376875; Santa Cruz Biotechnology). To amplify the signal, we used biotinylated horse anti-mouse/rabbit IgG antibody (BA-1400; Vector Laboratories, Newark, CA, USA) and streptavidin–horseradish peroxidase (SA-5004; Vector Laboratories). Immunoperoxidase staining was carried out using 3,3′-Diaminobenzidine tetrahydrochloride (DAB) (SK-4100, ImmPACT DAB Substrate kit, peroxidase, Vector Laboratories). For TRAP, counterstaining with Harris hematoxylin was performed; however, no counterstaining was applied for the other biomarkers (TNFα, IL-1β, VEGF, and OCN). As a negative control, the primary antibodies were omitted.

### 4.13. Analysis of the General Health Condition of the Animals and Intraoral Clinical Examination

Throughout the experimental period, the overall health of the animals was monitored, and any occurrences were recorded and analyzed. Body weight measurements were taken three times: before the extraction of the upper incisors and the installation of the DI, during the reopening and installation of the transmucosal component, and just before euthanasia. The results are expressed in grams (g) as mean ± standard deviation for each experimental group. A thorough intraoral clinical examination was conducted to inspect the surgical area or the peri-implant region at the same intervals mentioned.

### 4.14. Histopathological Analysis

The histopathological analysis was conducted by a calibrated examiner who was blinded to the study groups, and the results were subsequently validated by a certified histologist (E.E.). For all microscopic analyses, a light microscope (Carl Zeiss Microscopy GmbH, NI, ALE, Jena, Germany) equipped with a digital camera (also from Carl Zeiss Microscopy GmbH) was utilized, with the output connected to a computer. The photomicrographs of the histological slides were captured using ZEN2 software (Carl Zeiss Microscopy GmbH). The region of interest (ROI) designated for histopathological analysis, referred to as ROI-1, was defined as a rectangular area measuring 6264 × 4716 μm. This rectangle was oriented along the long axis of the implant and included all peri-implant tissues throughout the length of the implant (Figure 5). In this region, the following parameters were evaluated: cellularity pattern and structuring of peri-implant soft tissues; cellularity pattern and structuring of peri-implant bone tissue; presence, nature, and intensity of the local inflammatory response; and extent of the inflammatory process, when present.

### 4.15. Histometric Analyses of the Percentage of Total Bone Tissue (B.Ar/T.Ar) and the Percentage of Non-Vital Bone Tissue (NVB.Ar/B.Ar)

The histometric analyses were conducted by two calibrated examiners who were blinded to the group assignments. These analyses were performed separately and at two different times, with the arithmetic mean of their measurements being calculated. The measurements were taken using ImageJ^®^ software (version 1.5i; National Institutes of Health, Bethesda, MD, USA) with the aid of the Polygon Selections tool. The B.Ar/T.Ar analysis was carried out on the previously defined region of interest (ROI-1) following the parameters set forth by Souza et al. (2024) [63]. In summary, the analysis measured the percentage of the total image area occupied by bone tissue while subtracting the area occupied by the intervening space (Figure 5).

The NVB.Ar/B.Ar analysis was conducted on the region of interest (ROI) designated as ROI-2. This area was defined as a rectangle measuring 2784 × 2096 μm, with the longest side oriented along the long axis of the implant. The rectangle was positioned on the palatal side of the space occupied by the DI, encompassing its central coils. The proximal half of the rectangle included the coils and the peri-implant tissues immediately associated with them, while the distal half contained the pre-existing bone tissue in that region (Figure 5). The NVB.Ar/B.Ar was calculated based on the parameters established by Souza et al. (2024) [63]. In summary, the percentage of the total area of bone tissue in the image that consisted of non-vital bone tissue was measured. This non-vital bone tissue was characterized by the presence of contiguous lacunae that lacked osteocytes and/or contained only remnants of these cells. The results of both histometric analyses are expressed as percentages (%), presented as means ± standard deviations for each experimental group.

### 4.16. Immunohistochemical Analyses

Immunohistochemical analyses were conducted by a calibrated examiner who was blinded to the group assignments and were subsequently validated by a certified histologist. These analyses focused on two distinct regions of interest (ROIs), referred to as ROI-3 and ROI-4. ROI-3 was utilized to evaluate the immunolabeling of osteocalcin (OCN) and tartrate-resistant acid phosphatase (TRAP). This area was defined using two rectangular sections, each measuring 348 × 262 μm. The longer sides of the rectangles aligned with the long axis of the implant. These rectangles were placed in the bone tissue on the palatal and vestibular sides of the space occupied by the DI, positioned centrally in the alveolar process, with the alveolar bone crest serving as the coronal limit (Figure 5).

ROI-4 was used to analyze the immunolabeling of TNFα, IL-1β, and VEGF. This area included two rectangles, each measuring 348 μm by 262 μm, with their longer sides aligned along the long axis of the implant. The rectangles were positioned in the peri-implant mucosa region, specifically on the palatal and vestibular sides of the space occupied by the DI, with the supraperiosteal peri-implant connective tissue at their centre (Figure 7).

For the analysis of TNFα, IL-1β, VEGF, and OCN, immunolabeling was quantified using ImageJ^®^ software (National Institutes of Health). The Colour Threshold tool facilitated the measurement of immunolabeling density, and the results are presented as a percentage (%), expressed as the mean ± standard deviation for each experimental group.

For TRAP, we quantified both the total number of TRAP-positive cells and the number of TRAP-positive cells attached to the bone matrix using the same ImageJ software. The results are reported as the number of cells per mm^2^, also expressed as the mean ± standard deviation for each experimental group.

### 4.17. Statistical Analysis

The primary outcome of this study was the NVB.Ar/B.Ar. In contrast, the other analyses—including general health conditions, intraoral clinical examinations, histopathological analysis, immunohistochemical analyses, and B.Ar/T.Ar—were considered secondary variables. The statistical analysis of the quantitative data was conducted using BioEstat 5.3^®^ software (Instituto de Mamiruá, Tefé, AM, Brazil). To assess the normality of data distribution, the Shapiro–Wilk test was utilized. Following this, the Analysis of Variance (ANOVA) statistical test was performed, along with Tukey’s post hoc test. A significance level of 5% (*p* < 0.05) was adopted for the analyses. The sample size calculation aimed for a study power greater than 80% (with an alpha level of 5% and a type B error rate of 20%), based on a previous study that employed a similar methodology [63].

## 5. Conclusions

The results indicate that mechanical debridement, when used both conventionally and as a standalone treatment, was neither effective nor safe for treating experimental peri-implantitis induced by ligature in osseointegrated implants within the maxilla of aged rats treatment with zoledronate. Following treatment with an oncological dose of zoledronate, mechanical debridement resulted in a relative reduction in the inflammatory process. However, it also caused a significant increase in the amount of non-vital bone tissue, leading to cellular changes and alterations in the histophysiology of the peri-implant region. These factors may increase the risk of MRONJ-DI.

## Data Availability

The original contributions presented in this study are included in the article. Further inquiries can be directed to the corresponding author.
